# Complete chloroplast genome of *Herpetineuron toccoae* (Sull. & Lesq.) Cardot, a winter host of gall aphids inducing the formation of *Galla chinensis*

**DOI:** 10.1080/23802359.2026.2680779

**Published:** 2026-06-11

**Authors:** Qin-nan Zhou, Fa-xuan Zhang, Sheng-yi Chen, Hui Mu, Zhen-yan Yuan

**Affiliations:** a College of Ecology and Resources Engineering, Wuyi University, Wuyishan, PR China; b Fujian Provincial Key Laboratory of Eco-Industrial Green Technology, Wuyi University, Wuyishan, PR China

**Keywords:** *Herpetineuron toccoae*, Anomodontaceae, chloroplast genome, phylogeny, *Galla chinensis*

## Abstract

Bryophytes constitute a fundamental component of many ecosystems worldwide. In China, certain bryophyte species serve as key winter hosts for gall aphids that induce the formation of *Galla chinensis*, an important resource in traditional Chinese medicine. Here, we sequenced and characterized the complete chloroplast genome of *Herpetineuron toccoae* (Sull. & Lesq.) Cardot. The chloroplast genome is 124,934 bp in length and exhibits a typical quadripartite structure, comprising a large single-copy region (87,005 bp), a small single-copy region (18,465 bp), and two inverted repeats (9,732 bp each). The overall GC content is 28.5%. The genome encodes 126 genes, including 80 protein-coding genes, 37 transfer RNA genes, eight ribosomal RNA genes, and one pseudogene. Phylogenetic analysis placed *H. toccoae* within a clade of pleurocarpous mosses with high bootstrap support. This chloroplast genome provides a valuable resource for phylogenetic and evolutionary studies of the genus *Herpetineuron*.

## Introduction

**Figure 1.**
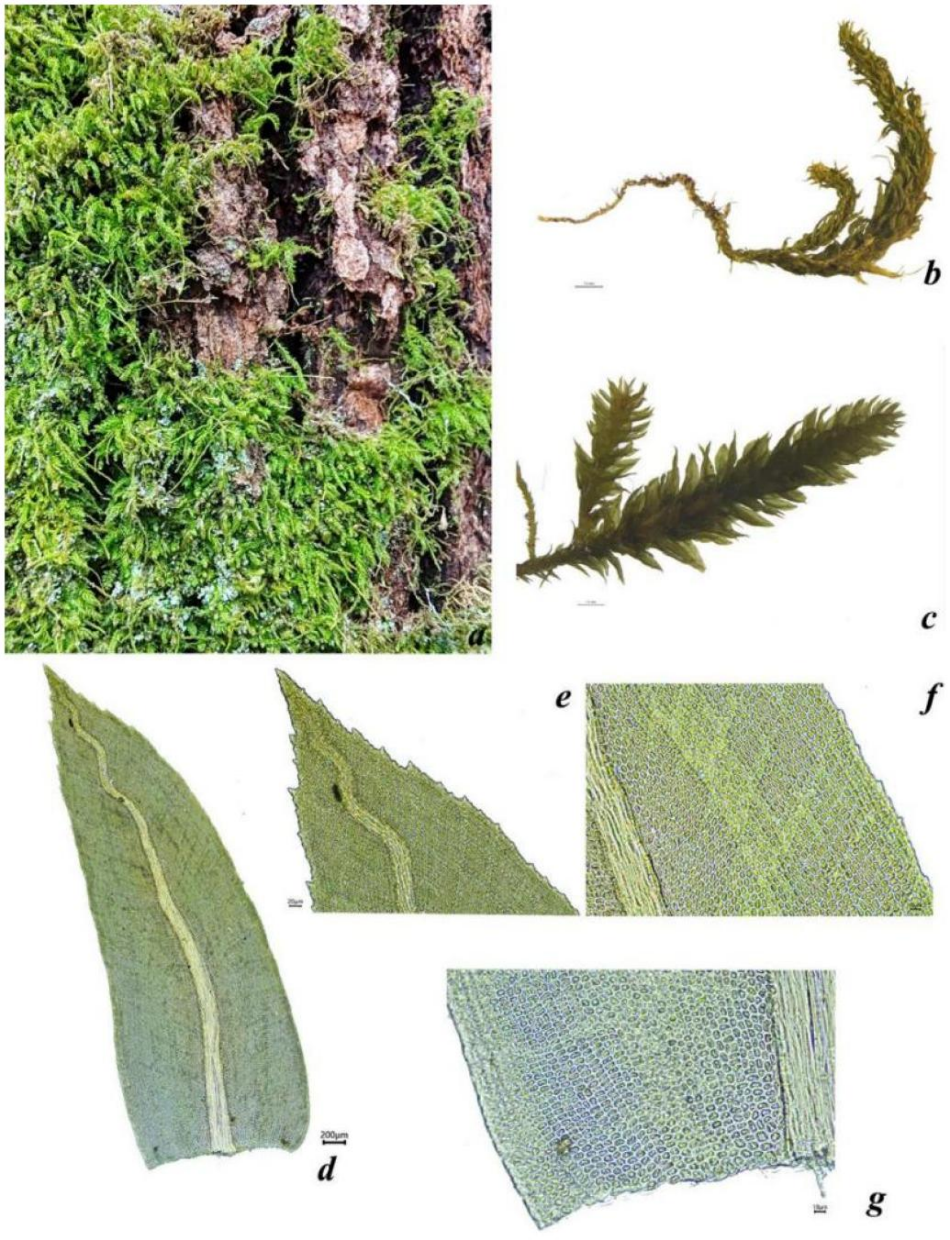
Photograph of *Herpetineuron toccoae* (Sull. & Lesq.) Cardot (photographed by Zhen-Yan Yuan in the Wuyi Mountains, China). Plants are small and delicate and grow in damp, shaded habitats. Stems are thick and generally branched, and are terete-foliate when dry. Leaves are small and lanceolate, becoming involute and tubular when dry, with a broadly decurrent base. Marginal teeth comprise one to several cells, and the costa is sinuous in the distal one-third. (a) Plant. (b) Plant when dry. (c) Plant when wet. (d) Leaf. (e) Apical region of the leaf. (f) Middle region of the leaf. (g) Leaf base.

*Herpetineuron toccoae* (Sull. & Lesq.) Cardot (Cardot 1905) is a moss species in the genus *Herpetineuron* (Müll. Hal.) Cardot (Anomodontaceae). Morphologically, it is medium-sized to robust, with rigid growth forming dense, interwoven tufts that range from yellowish green to brownish green. Primary stems are creeping and frequently produce asexual stoloniferous branches. Secondary stems are erect or ascending, with irregular to sparse branching. Stem apices are circinate when dry and caudate when moist. Leaves on stems and branches are similar, ovate-lanceolate, and terminate in an acuminate apex. Leaf margins are entire near the base but become irregularly and coarsely dentate toward the apex. The costa is strong, gradually tapering, and distinctly zigzag near the leaf tip (Wu et al. 2002) (Figure 1). Ecologically, *H. toccoae* is widely distributed and exhibits strong adaptability. In China, it occurs across diverse habitats and ecological settings. Notably, *H. toccoae* serves as the winter host for *Melaphis chinensis* (Bell) Baker, the gall-forming aphid responsible for inducing *Galla chinensis*, an important resource in traditional Chinese medicine. During the summer phase of the aphid life cycle, galls develop on hosts in the family Anacardiaceae rather than on *H. toccoae* (Liu et al. 2023).

Galla chinensis, also known as Chinese gallnut, is a specialized gall induced by aphids in the family Pemphigidae. These aphids parasitize and feed on the leaves of host plants such as *Rhus chinensis* Mill. (Anacardiaceae) and require bryophytes as winter hosts. *G. chinensis* is a well-documented medicinal material with diverse pharmacological activities. As one of the three key elements in *G. chinensis* production, bryophytes (the winter hosts of gall-forming aphids) are primarily collected from wild populations. The chloroplast genome dataset generated here will support future genetic studies aimed at the conservation and sustainable utilization of bryophytes that serve as hosts for these aphids.

## Materials and methods

Plant samples of *H. toccoae* were collected from the Wuyi Mountains (117.940000°E, 27.790833°N), Fujian Province, China. The voucher specimen was deposited in the Herbarium of Wuyi University under accession number WY02 (www.wuyiu.edu.cn; Zhen-Yan Yuan; 65482398@qq.com). Fresh material was sent to Nanjing Genepioneer Biotechnology Co., Ltd. (Nanjing, China) for genomic DNA extraction using a plant genomic DNA kit (Tiangen Biotech, Beijing, China), followed by library construction. Sequencing was performed on an Illumina NovaSeq 6000 platform (Illumina, San Diego, CA). The chloroplast (cp) genome was assembled using GetOrganelle (Freudenthal et al. 2020). The minimum, maximum, and mean read-mapping depths for the assembled genome were 107×, 2491×, and 462.43×, respectively (Fig. S1). Chloroplast genome annotation was performed using two approaches. First, protein-coding sequences (CDSs), rRNAs, and tRNAs were predicted using Prodigal v2.6.3 (Hyatt et al. 2010), HMMER v3.1 (Potter et al. 2018), and ARAGORN v1.2.38 (Laslett and Canback 2004), respectively. Second, the chloroplast genome of *Thuidium* sp. (GenBank accession number MW429506.1) was used as a reference for BLAST-based annotation (BLAST v2.6). The two annotation results were integrated, and gene boundaries were manually curated. Cis- and trans-splicing maps for *H. toccoae* were generated using CPGview (Figure S2) (Liu et al. 2023). The annotated chloroplast genome has been deposited in GenBank (NCBI) under accession number PV138164.1. A circular chloroplast genome map was generated using OGDRAW (Tillich et al. 2017). The plastome sequences were aligned using MAFFT v7.490 with default parameters. Unreliable regions in the alignment were removed using trimAl v1.4.rev15. Based on the Bayesian Information Criterion, the optimal nucleotide substitution model was selected using jModelTest v2.1.10. To determine the phylogenetic position of *H. toccoae*, a maximum-likelihood (ML) phylogeny was inferred from complete chloroplast genomes of 25 species using RAxML v8.2.10 (Stamatakis 2014).

## Results

**Figure 2.**
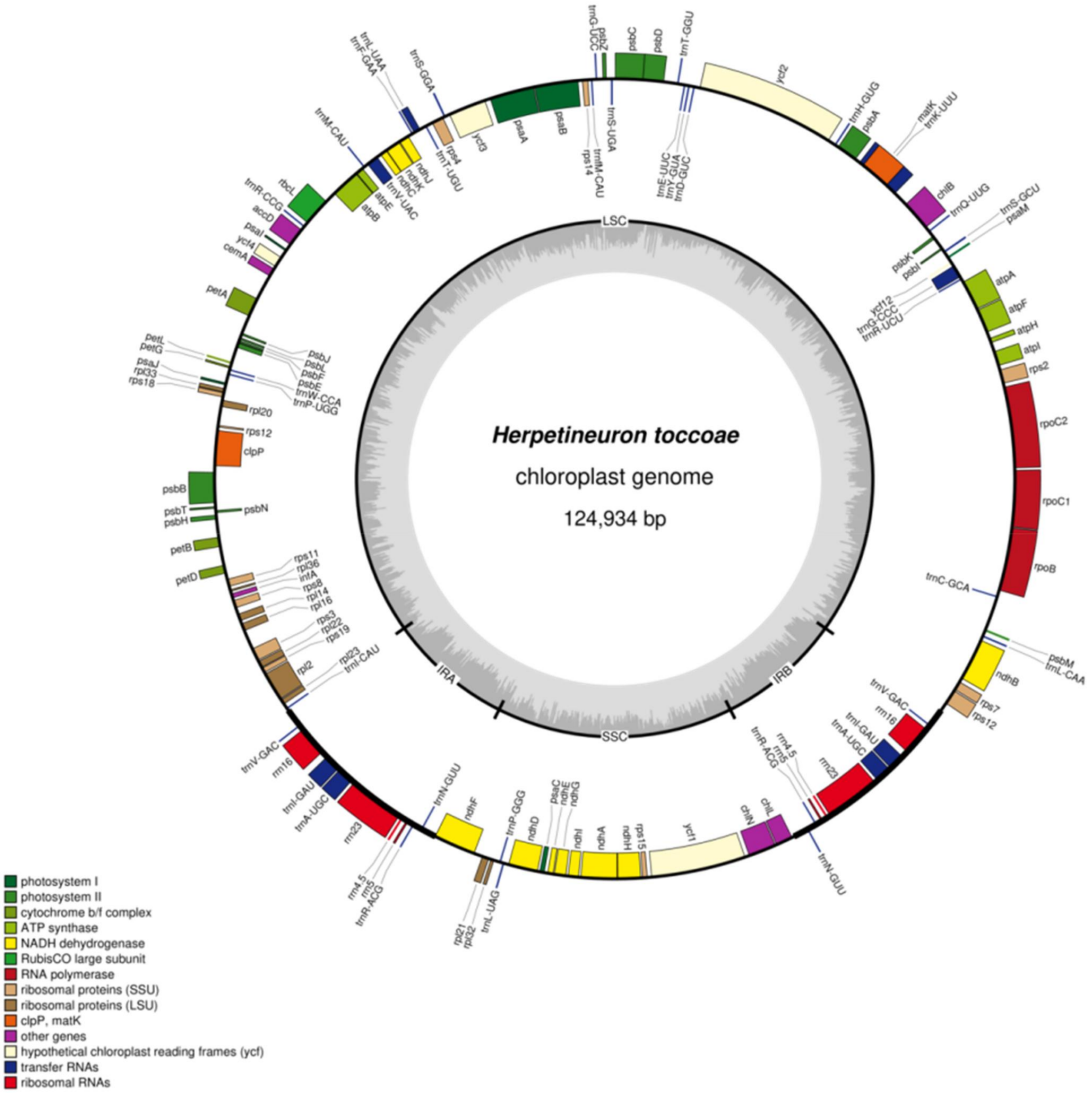
Circular map of the *Herpetineuron toccoae* (Sull. & Lesq.) Cardot chloroplast genome. Boxes on the outer circle represent genes, with box size corresponding to gene length. Genes transcribed clockwise and counterclockwise are shown on the outer and inner sides of the circle, respectively. The inner circle indicates GC content (dark gray) and AT content (light gray). Thick lines denote the inverted repeat regions (IRa and IRb), which separate the LSC and SSC regions.

The complete chloroplast genome of *H. toccoae* is 124,934 bp in length (GenBank accession number PV138164.1) (Figure 2). It exhibits a typical quadripartite structure, comprising a large single-copy (LSC) region (87,005 bp) and a small single-copy (SSC) region (18,465 bp), separated by two inverted repeats (IRs) of 9,732 bp each. The nucleotide composition is 35.7% A, 14.2% C, 14.3% G, and 35.7% T, corresponding to an AT content of 71.4% and a GC content of 28.5%. The genome encodes 126 genes, including 80 protein-coding genes, 37 tRNA genes, eight rRNA genes, and one pseudogene. Additionally, two genes (ycf3 and clpP) each contain two introns. The trans-spliced gene rps12 comprises three exons located in the LSC region (Figure S2).

**Figure 3.**
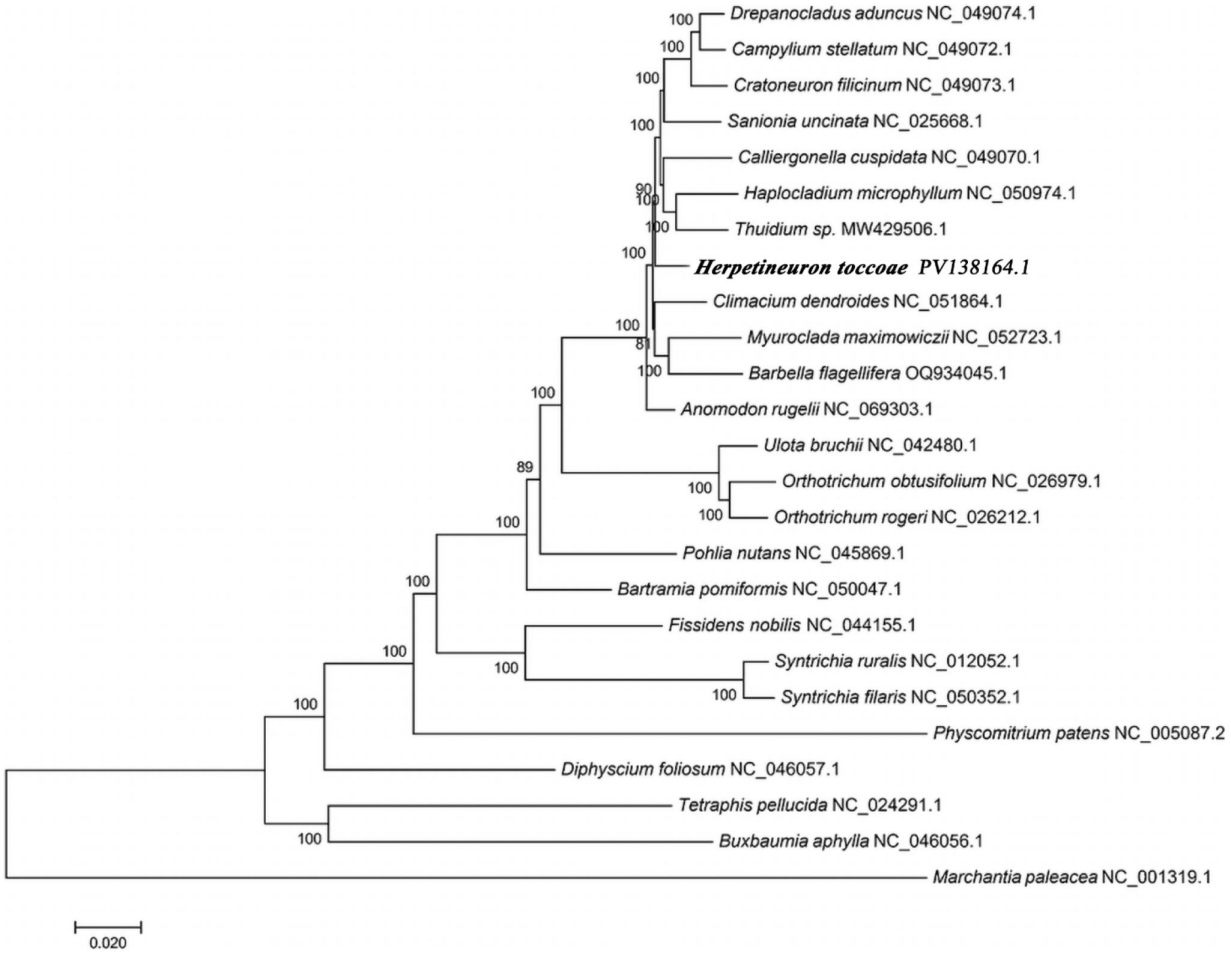
Maximum-likelihood phylogeny of *Herpetineuron toccoae* and other bryophytes inferred from complete chloroplast genome sequences, with *Marchantia paleacea* (NC_001319.1; Dong et al. 2019) as the outgroup. Numbers on branches indicate ML bootstrap support values. Sequences used for tree construction: *A. rugelii* (NC_069303.1), *B. aphylla* (NC_046056.1), *B. flagellifera* (OQ934045.1; Yuan et al. 2024), *B. pomiformis* (NC_050047.1), *C. cuspidata* (NC_049070.1; Sheng et al. 2020), *C. dendroides* (NC_051864.1; Han et al. 2020a), *C. filicinum* (NC_049073.1; Sheng et al. 2020), *C. stellatum* (NC_049072.1), *D. aduncus* (NC_049074.1), *D. foliosum* (NC_046057.1), *F. nobilis* (NC_044155.1; Kwon et al. 2019), *H. microphyllum* (NC_050974.1), *H. toccoae* (PV138164.1), *M. maximowiczii* (NC_052723.1; Han et al. 2020b), *O. obtusifolium* (NC_026979.1), *O. rogeri* (NC_026212.1), *P. nutans* (NC_045869.1; Jin et al. 2020), *P. patens* (NC_005087.2), *S. uncinata* (NC_025668.1; Park et al. 2018), *S. filaris* (NC_050352.1; Kim et al. 2019), *S. ruralis* (NC_012052.1), *T. pellucida* (NC_024291.1; Bell et al. 2014), *thuidium* sp. (MW429506.1), and *U. bruchii* (NC_042480.1).

A phylogenetic tree was constructed using the complete chloroplast genome of H. toccoae, along with 23 complete chloroplast genomes from Bryophyta and one from Marchantiophyta retrieved from NCBI. The chloroplast genome of *Marchantia paleacea* was used as the outgroup (Figure 3). ML inference was performed in RAxML under the GTR+GAMMA model with 1000 bootstrap replicates to assess branch support. The ML analysis indicated that *H. toccoae* and *Anomodon rugelii* (Anomodontaceae) formed two distinct, well-supported clades. *H. toccoae* was most closely related to *Haplohymenium* and *Thuidium*.

## Discussion and conclusion

In this study, we assembled the complete chloroplast genome of *H. toccoae* using Illumina sequencing technology. The *H. toccoae* chloroplast genome is 124,934 bp in length and exhibits a typical quadripartite structure, encoding 80 protein-coding genes, 37 tRNA genes, eight rRNA genes, and one pseudogene. Gene content and organization are highly conserved and similar to those of other bryophyte chloroplast genomes. Our annotation revealed a rare arrangement of the rps12 gene, with all three exons located in the LSC region. This contrasts with the canonical trans-splicing model observed in most land plants, where exon 1 is situated in the LSC region, and exons 2 and 3 reside in the IR region. A maximum-likelihood phylogenetic tree indicated that *H. toccoae* is most closely related to *Haplohymenium* and *Thuidium*. In analyses based on nuclear ribosomal DNA internal transcribed spacers (ITS), *Anomodon*, *Herpetineuron*, and *Haplohymenium* were recovered within a single clade (Zhang et al. 2003). More recent phylogenetic analyses incorporating nuclear ITS, plastid trnS–F, rpl16, atpB–rbcL, and mitochondrial nad5 regions have indicated polyphyly within *Anomodon* and support its segregation into four genera (Michae et al. 2019). Our chloroplast genome-based ML results are consistent with these studies and further support the proposed relationships among these taxa. Overall, this work reports the first complete chloroplast genome for *H. toccoae* and provides a resource for studying moss evolution, particularly within the family Anomodontaceae. In addition, characterization of genetic resources in moss host species associated with insect galls may support the development of artificial propagation and breeding strategies.

## Supplementary Material

Supplementary Figure S1.docx

Supplementary Figure S2.docx

## Data Availability

The genome sequence data supporting this study are available in GenBank (NCBI) under accession number PV138164. The associated BioProject, SRA, and BioSample identifiers are PRJNA1212882, SRR32087719, and SAMN46312239, respectively.
