# Supplementary material for: Complete chloroplast genome of Herpetineuron toccoae (Sull. & Lesq.) Cardot, a winter host of gall aphids inducing the formation of Galla chinensis
Source: Mitochondrial DNA B Resour. 2026 Jun 11;11(7):859–63. doi: 10.1080/23802359.2026.2680779 (PMC13262100; doi:10.1080/23802359.2026.2680779)
Supplement: Supplementary Figure S1.docx [file TMDN_A_2680779_SM6789.docx]

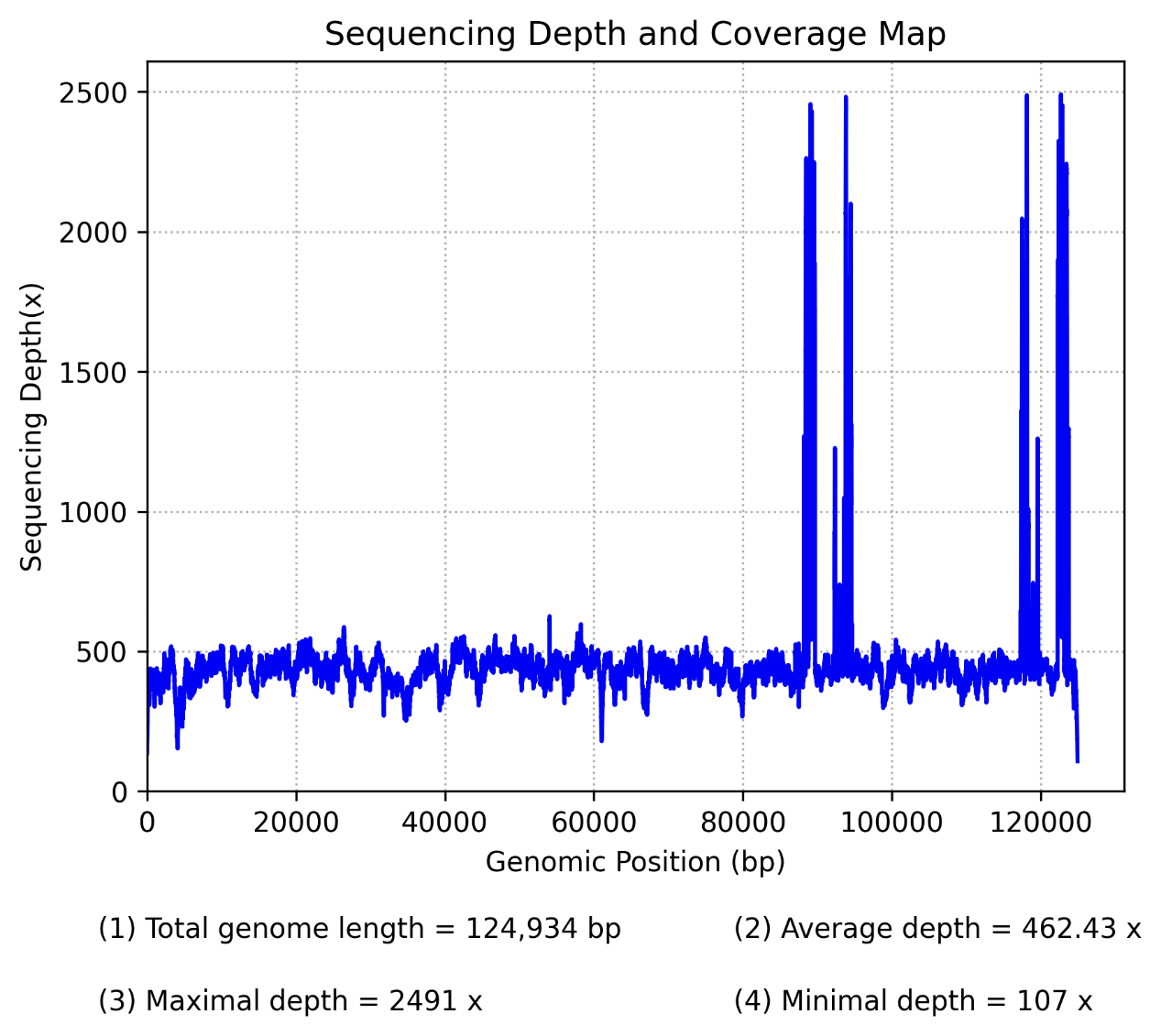


Supplementary Figure S1. Map of Sequencing Depth and Coverage. The mapping depth of clean reads is represented by blue bars. The X-axis and Y-axis correspond to the genomic position of the plastome and sequencing depth, respectively. Specifically, the maximum sequencing depth reaches ×2491, the minimum depth is ×107, and the average depth is ×462.43.
