# Supplementary material for: Complete chloroplast genome of Herpetineuron toccoae (Sull. & Lesq.) Cardot, a winter host of gall aphids inducing the formation of Galla chinensis
Source: Mitochondrial DNA B Resour. 2026 Jun 11;11(7):859–63. doi: 10.1080/23802359.2026.2680779 (PMC13262100; doi:10.1080/23802359.2026.2680779)
Supplement: Supplementary Figure S2.docx [file TMDN_A_2680779_SM6788.docx]

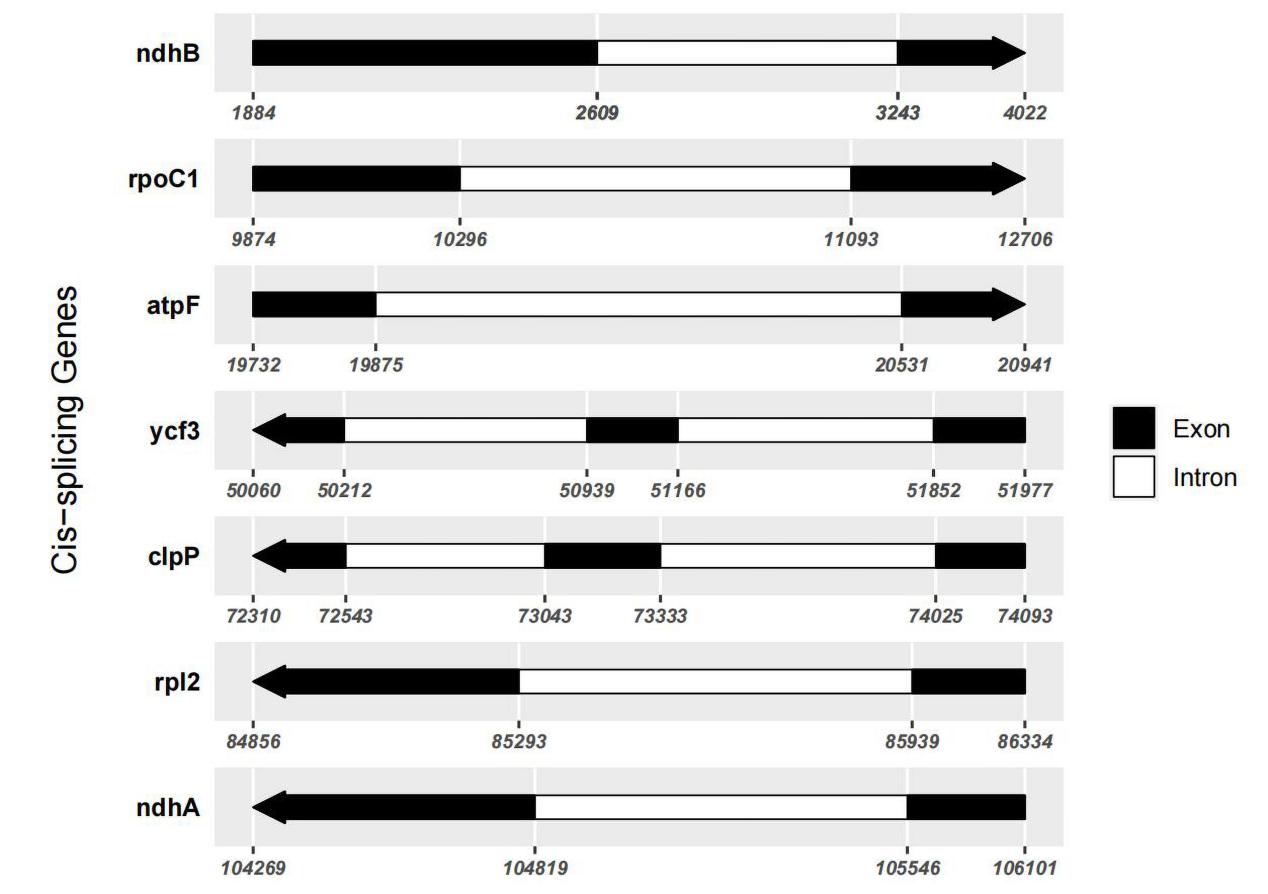


（a）


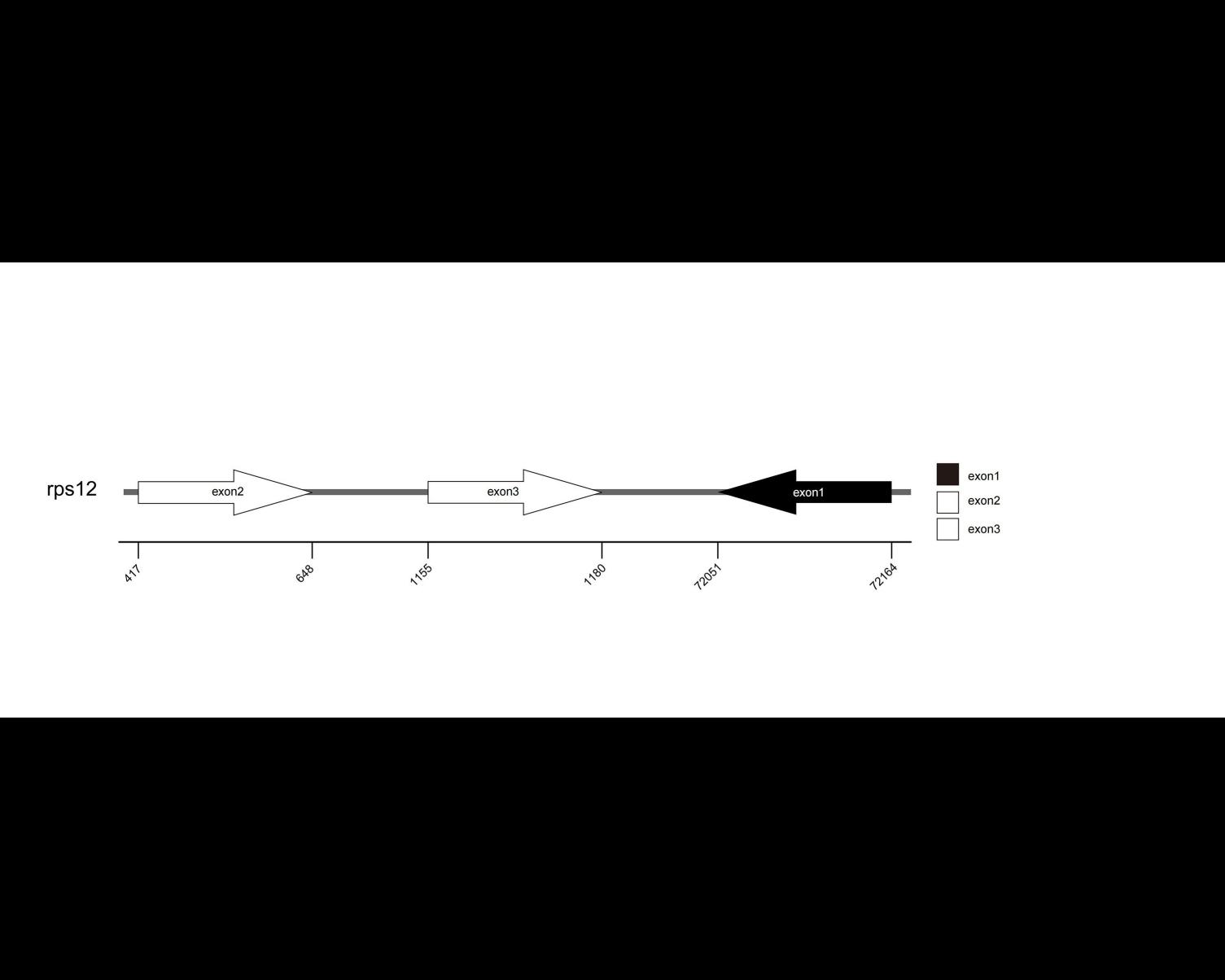


（b）

Supplementary Figure S2.(a) Schematic diagram of cis-splicing genes in the chloroplast genome of *Herpetineuron toccoae*.Genes are arranged from top to bottom according to their order in the chloroplast genome, with gene names labeled on the left and gene structures shown on the right,and gene structures are shown on the right; exons are indicated in black, introns in white, and arrows indicate the gene's sense direction. Supplementary Figure S2.(b) Schematic diagram of trans-splicing genes in the chloroplast genome of *Herpetineuron toccoae.* rps12 gene is located in the LSC region and contains three exons。The first exon (72164–72051) is located near the LSC boundary，the second exon (417–648) and the third exon (1155–1180) are separated in the genome.
